# Social Network Analysis of Multi-level Linkages: A Swedish Case Study on Northern Forest-Based Sectors

**DOI:** 10.1007/s13280-014-0492-0

**Published:** 2014-02-26

**Authors:** E. Carina H. Keskitalo, Julia Baird, Emmeline Laszlo Ambjörnsson, Ryan Plummer

**Affiliations:** 1 Geography and Economic History, Umeå University, 901 87 Umeå, Sweden; 2 Environmental Sustainability Research Centre, Brock University, St. Catharines, ON L2S 3A1 Canada; 3 Stockholm Resilience Centre, Stockholm University, Kräftriket 2B, 114 19 Stockholm, Sweden

**Keywords:** Forest, Social network analysis, Multi-level, Interaction, Sweden

## Abstract

Forest use in Northern Sweden is being influenced both by global trends and local situations. This results in interactions between numerous groups that may impact local forest governance. Social network analysis can here provide insight into the total pattern of positive, negative, and cross-level interactions within user group community structure (within and among groups). This study analyses interactions within selected renewable resource sectors in two northern Swedish municipalities, both with regard to whether they are positive, neutral, or negative, as well as with regard to how local actors relate to actors across levels, e.g., with regional, national, and international actors. The study illustrates that many interactions both within and outside a given sector are seen as neutral or positive, and that considerable interaction and impact are defined as national and in some cases even international. It also indicates that the impact of Sweden’s only existing Model Forest may to some extent constitute a bridge between different sectors and levels, in comparison with the interactions between sectors in a municipality where such a cooperation mechanism does not exist.

## Introduction

Northern Sweden has a long tradition of renewable and non-renewable resource industries, with industries today ranging from major multinational companies of domestic or international origin to single-person entrepreneurs in multiple sectors. In northern Europe, interests such as mining, wind power, forestry, tourism, recreation, hunting, fishing, environmental protection, and reindeer husbandry often use the same or adjacent areas within, for instance, a single local government (municipal) jurisdiction. Conflicts between and among these uses are well described in literature and often concern the potential infringement of one interest under establishment on an existing industry or business in an area. Examples include the establishment of wind power or environmental protection and its potential impacts on existing uses, such as the area available for forestry (in the case of environmental protection), or the possibilities for reindeer herding including grazing and migration (Keskitalo 2008a; Keskitalo et al. 2009; Söderholm and Pettersson 2011). Interests that are dispersed or occur over large areas are most likely to be impacted by any new establishment: research highlights in particular the limited possibilities for reindeer husbandry as a very small sector in contrast with the much larger forestry sector, which is conducting logging in areas used by reindeer husbandry for grazing or migration (e.g., Keskitalo 2008b; Sandström et al. 2008).

Rural areas of Northern Sweden are also subject to larger societal trends such as urbanisation, fragmentation of nature areas (e.g., due to well-developed road networks and other societal structures), and increasing pressure on resources (e.g., Nordlund and Westin 2011). As areas with old-growth forest have diminished, environmental protection interests have increased, including formal protection and administration of protected areas (Johansson and Lidestav 2011). Large economic differences between sectors as well as differences in terms of the size of economic actors thus exist. Property owners in these rural areas often have their main residence elsewhere. Many small-scale forest owners do not live on their holdings and gain their main income from other employment, and even most self-acknowledged Saami (the only group with general right to practice reindeer husbandry in Sweden) neither practice reindeer husbandry nor live in the traditional reindeer husbandry areas (cf. Keskitalo 2008b).

As employment in traditional sectors such as forestry has decreased (while maintaining or increasing forestry production due to substituting employment with technology), a larger emphasis has been placed on developing local employment sources to supplement forestry. In many areas (in Sweden as well as other countries), there has been a focus on tourism as such an industry, in line with a larger focus on service economies generally in the advanced industrialized (OECD) states. A focus has here often been placed on small-scale tourism, even if large tourism facilities may more easily be able to attract tourism for specific areas and compete in the international market (e.g., Müller and Ulrich 2007).

Forest use is thus impacted by both local situations and global trends, including multiple actors of different sizes and often in some conflict over use. This paper investigates *the existence of user interactions and impacts in the forest*-*based sectors* of forestry, reindeer husbandry, small-scale winter tourism, and environmental protection *at a local level, as well as how they extend to regional, national, and international levels.* Taking as its point of departure two Swedish municipalities with different internal cooperation structures, this study employs the lens of social network analysis. In addition to describing networks of governance in this context, the research explores specific network characteristics in relation to governance outcomes and challenges. The study illustrates that most interactions both within and outside given sectors are seen as neutral or positive, and that significant interactions and impacts occur at national and in some cases even international levels. It also shows that the impact of Sweden’s at the time of study only existing Model Forest may—in comparison to interactions between sectors in a municipality lacking such cooperation mechanisms—to some extent constitute a bridge between different sectors as well as between different levels of organization.

## Theoretical Framework

The investigation is conceptually positioned in relation to the growing literature on environmental governance. At a broad level, environmental governance refers to “the set of regulatory processes, mechanisms and organization through which political actors influence environmental actions and outcomes” (Lemos and Agrawal 2006, p. 298). The growing discourse on environmental governance is unfolding along multiple interconnected dimensions. One dimension addresses the rich plurality of models or modes of governance in reference to the environment. Lemos and Agrawal (2006), for example, identify the emergence of mechanisms and strategies (e.g., private-social partnerships, public–private partnerships, and co-management) at the intersections of the conventional social roles held by markets, communities, and states. Similarly, approaches also identify very different ways in which scale may be constructed: for instance, what may be regularly identified as a local scale of interaction may for instance be constructed by higher-level frameworks and also by interactions that are supralocal (Norman and Bakker 2009).

An excessively static and structural view of governance may thus be limiting in light of global environmental change (Plummer and Armitage 2010), with a more fruitful approach being to research the particular interactions between groups and how these manifest. Multi-level governance has been defined as the public and private actors on various levels that take part in decision-making (e.g., Marks and Hooghe 2004). This approach, developed to conceptually understand the role of the European Union (EU) in relation to national policy, is now broadly applied to understand not only EU but also private market as well as non-governmental organizations (NGOs) influences on the state (ibid). As the economic and political aspects of globalization—for instance impacting the privatization of services, hollowing-out of the state, and re-distribution of authority from national to supranational and subnational levels—have become more pronounced, multi-level governance is increasingly utilized to emphasize that government, although most often a crucial actor, is impacted by numerous other actors exercising decision-making and steering powers, including at a supranational level (Hooghe and Marks 2001). Thus, while local participation has often been seen as a suitable way to proceed on resource conflicts, and the local perspective on resource use has also been pronounced in the literature on adaptation to global climate change (e.g., Hovelsrud and Smit 2010, ed), regulatory frameworks on different levels—aside from direct resource rights—can be expected to have a large impact on local resource use. These regulatory frameworks may include decision-making structures established at the EU level, and national structures for regional and municipal administration that determine the ways in which interaction is developed, and for instance the degree of decentralisation of decision-making. A large impact may also result from informal norms of cooperation and the influence of different actors, which may not only characterize the interaction in sectors at large but also influence specific local-level decision-making (Keskitalo et al. 2009). In forestry, for instance, multi-level governance can be used to describe the impact of international and national norm development and market governance through forest certification systems (FSC and PEFC), often seen as having strong implementation of a requirement level above the law and in practice constituting a “market-based regulation” system (e.g., Johansson and Lidestav 2011).

This development results in an increasingly open question as to how actors at the local level actually include other levels, perceive impacts from other levels, and, in effect, participate in broader governance networks. Studies of networks may illustrate the role that higher levels—as well as sectors other than those reviewed—may play in what have often been assumed as relatively local practices. Viewing governance through a network lens may thus illuminate the formal and informal social structures of how governance is operationalised. Network analysis is a research approach used to understand the structure of a system using connections among nodes (e.g., actors, events, or other phenomena) and node attributes as a basis for analysis, and is broadly applied across several fields of study (Janssen et al. 2006). In natural resource governance, specific network features have been identified that tend to promote or inhibit governance through the diversity, density, and nature of connections across actors and scales (e.g., Newman and Dale 2005; Crona and Bodin 2006; Prell et al. 2009) and the role of individuals and groups of actors within the network (e.g., Bodin and Crona 2009; Hahn 2011). In forestry and forest-based natural resource governance in particular, social network analysis has been applied to investigate the impact of social connectivity on management of forest patches (Bodin and Tengö 2012), to analyze the implications of people’s relationships with forests on land use planning (Harshaw and Tindall 2005), and to evaluate forestry research networks (Klenk et al. 2009), among others.

Social network analysis provides the opportunity to evaluate patterns of interactions among actors or groups of actors operating within and across scales (e.g., Weiss et al. 2012; Baird et al. submitted) where challenges are pervasive and boundary or bridging organizations may act as intermediaries (Cash et al. 2006). Specific network attributes, such as actor diversity and cross-boundary and cross-scale interactions, have been identified as network properties that contribute to the adaptive capacity of environmental governance approaches (Sandström and Carlsson 2008; Bodin and Crona 2009; Sandström and Rova 2010). Accordingly, the role of individual actors in bridging boundaries and levels has been highlighted as an important attribute in natural resource governance (Lauber et al. 2008; Bodin and Crona 2009; Ernstson et al. 2010). Lauber et al. (2008), in a comparative case study of three successful natural resource management approaches, identified bonding (links between like groups) and bridging linkages (links between different groups) between local and non-local stakeholders as important attributes for success. Further, Bodin and Crona (2009), in a review of social networks in natural resource governance, highlighted the potential benefits of connectivity among sub-groups (e.g., different actor groups within and across scales) including greater likelihood of accessing diverse information and adoption of new practices, as well as of facilitating collective action that may not have otherwise occurred. Ernstson et al. (2010) highlighted the important role of scale-crossing brokers whose perspective is focused on the scale of the ecosystem for linking local and broad-scale (e.g., national, international) actors in ecosystem governance. Similarly, Lauber et al. (2008) found that interactions at the scale of the region being governed were most important for idea exchange and the development of consensus around a management plan.

The quality of interactions is another important governance attribute that may be investigated by network analysis. Positive interactions have often been emphasized in research, but neutral interactions are also important: such interactions may indicate for instance everyday interactions that may not be attributed any specific particularly negative or positive value, but may be crucial for conducting work and building relationships. These interactions can be considered as “strongly positive” and “weakly positive,” respectively (Labianca and Brass 2006). Negative interactions or relationships, however, are rarely examined in network analysis (Labianca and Brass 2006; Ansell 2008; Bruggeman et al. 2012), but may offer a richer perspective by identifying interactions that influence user group community structure (within and among groups) (Bruggeman et al. 2012) and may inhibit or jeopardize governance efforts (Jones et al. 1997; Tucker 2004; Robins et al. 2011). In fact, negative interactions are argued to have a greater impact on networks than positive or neutral interactions (Labianca and Brass 2006).

## Case Study and Methods

The boreal forest landscape with multiple uses is characteristic of northern Sweden. In this landscape forestry and reindeer husbandry are practiced largely on the same land areas, and any other users in these areas have some impact on, or relation to, these two sectors. Forestry constitutes an economically nationally important sector (about 10 % of Sweden’s export value or 3 % of GDP) relevant to large-scale interests as well as to a large number of small-scale forest owners (Johansson 2013; Swedish Forest Agency 2013a, b). Large forest companies, non-industrial private forest owners and government own, respectively, about 50, 38, and 6 % of forested land in the three northernmost counties of Sweden and constitute some 60 000 owners in total (Sandström and Widmark 2007). Reindeer husbandry, on the other hand, while practiced over more than a third of the Swedish land area (Government Offices of Sweden 2013),^1^ is a relatively small-scale industry, practiced by some 2500 reindeer herders active largely as individual entrepreneurs within reindeer husbandry units (Swe. *sameby*). In comparison with forestry, reindeer husbandry has negligible economic importance in a national context, but is important in certain local cases as well as to Saami indigenous identity (self-identified as some 20 000 people in Sweden in total) (cf. Keskitalo 2008b; Ministry of Agriculture 2009; Ministry for Rural Affairs 2013; Saami Parliament 2013).^2^ As reindeer husbandry constitutes a user right, it is largely practiced on lands owned by forest owners (large-scale owners as well as small holders and municipalities). Property rights in the area are thereby twofold, consisting of both an ownership right (on the part of forest or other land owners), and a user right (on the part of reindeer husbandry).

Tourism differs in its extent of development in the study areas, with some notably large-scale tourism destinations and facilities constituting the largest economic impact, and small-scale tourism, here viewed in terms of winter tourism, often in a relatively small or in the initial stages of development (Müller and Ulrich 2007). Tourism may, however, draw on, and benefit from, the practices of other resource sectors (e.g., forest roads offer accessibility for tourism locations). With regard to environmental protection, relatively large areas particularly in the inland municipalities targeted in this study are protected (Statistics Sweden 2008). Different from the case for instance for small-scale tourism, many sectors may view environmental protection as competition for use of resources. All of the above-mentioned renewable resource-based practices as well as non-renewable forest-based practices also interact among other land uses through the framework (boundaries and demands) of existing regulation and cooperation-based requirements (such as forest certification).

**Fig. 1:**
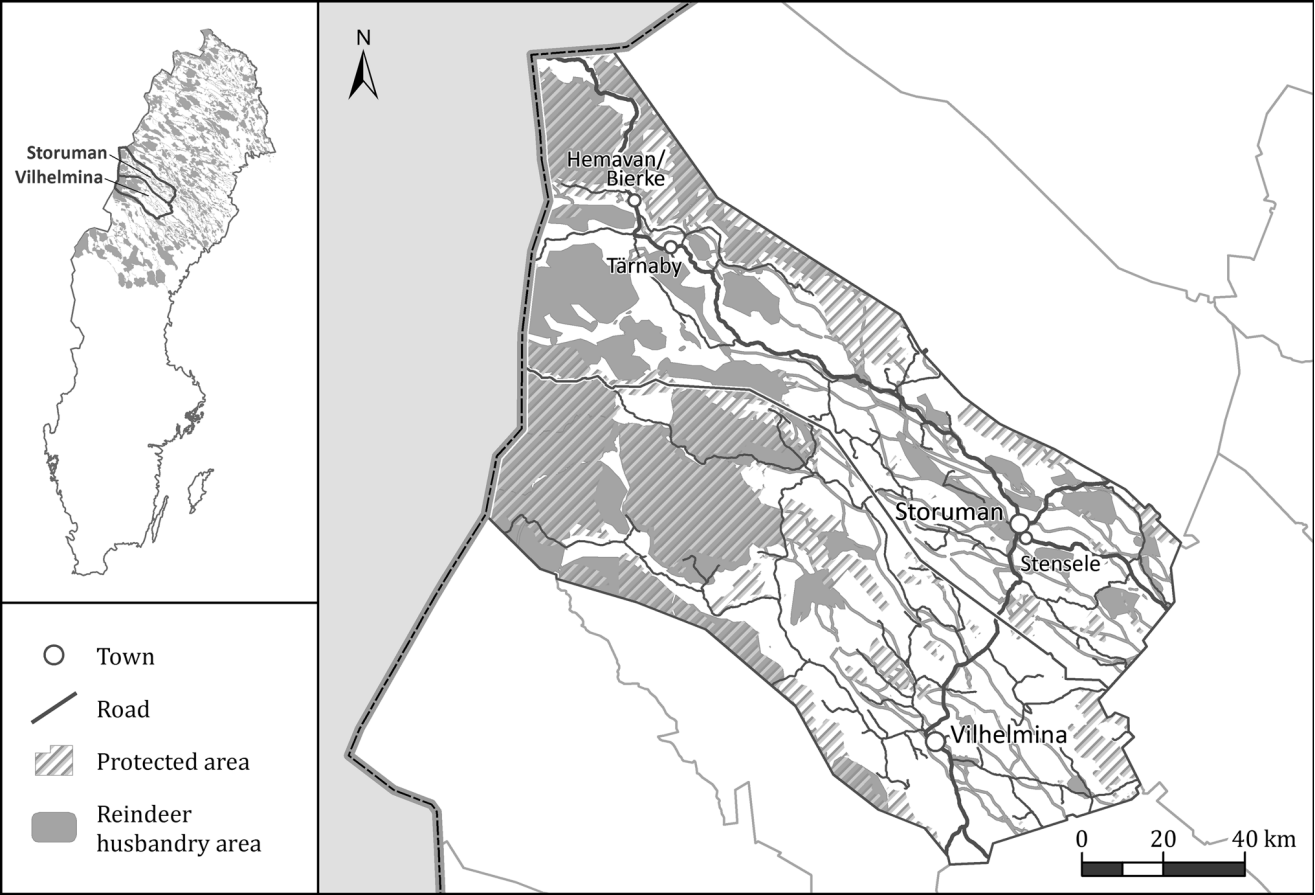
Case study locations and attributes

In this paper, multi-level governance concepts are drawn upon in order to: conceive of the potential interactions both across sectors and across levels (local, regional, national, and international); and, describe interactions in terms of the nature (i.e., the tone of the interaction: positive, neutral or negative) and type (i.e., interactions within or among sectors, public and private interests, and cross-level). The interactions and perceived impacts between actors in these four sectors as well as other sectors identified by study participants are considered in two neighboring inland municipalities, Vilhelmina and Storuman. These neighboring municipalities were chosen to investigate potentially varying levels of formal cooperation developed in forests, in particular in relation to the development of multi-use forest cooperation. Vilhelmina municipality (pop. approx. 7000) holds Sweden’s only (at the time of study) acknowledged Model Forest, Vilhelmina Model Forest. This development was inspired by the Canadian Model Forest concept forwarded at the UNCED in Rio in 1992 to support cooperation between different forest users. The aim of the model forest concept is to encourage multi-level interaction and network development associated with forest use, and may as such potentially increase interactions among actors. However, large variations may exist in how different model forests develop, given local preconditions and resources. Vilhelmina Model Forest is, for instance, more limited in its funding and staff than Canadian examples such as the Prince Albert Model Forest in Saskatchewan (see, e.g., Prince Albert Model Forest 2010). The neighboring municipality to Vilhelmina, Storuman (pop. approx. 6000) is relatively structurally similar to Vilhelmina in terms of the existence of forestry, reindeer husbandry, small-scale winter tourism, and environmental protection interests, although of course local differences particular to the municipality may exist. However, Storuman does not include model forest developments or specific formal cooperation structures other than what is common in any rural forest municipality (Fig. 1).

The local studies draw upon 54 semi-structured interviews conducted in Vilhelmina and Storuman during 2009. Two interviews (one for reindeer husbandry and one for authorities) covered both locations and thus were coded twice. In total, 28 interviews were coded for Vilhelmina and 26 for Storuman, resulting in 56 coded responses. All interviews were transcribed in full as a basis for the analysis. Selection of interviewees targeted local companies identified in each of the four focal sectors: forestry, reindeer husbandry, small-scale winter tourism, and environmental protection, as well as authorities relevant to these on local and regional levels. Interviewee organizations in the different sectors were selected based on their inclusion in official registers (e.g., of economic actors in the municipalities) and thus comprise the main actors active at the time in the selected sectors and municipalities. Interview questions targeted interviewees’ perceptions of factors that impacted their businesses, including factors impacting environmental conditions. Specific sections targeted interaction with and impact of different actors, with interviewees being encouraged to discuss interactions with different groups and actors in their responses to the interview questions. The social network analysis undertaken is based upon these responses. However, it should be noted that interviewee statements were likely to focus more on actors with whom they had frequent or perceived important interactions, thus the responses do not constitute a full, complete listing of all existing interactions.

In the social network analysis, interactions were coded for each interviewee based on his or her identified interactions with other individuals, organizations, or sectors, creating an ego network for each interviewee. These networks were combined to provide a perspective of the network of interactions among forest users. The context within which each interaction was mentioned was coded as: (i) positive, where the interviewee indicated a positive or good relationship; (ii) neutral, where the interaction was mentioned with no positive or negative association; or (iii) negative, where the interviewee indicated a dislike or negative relationship. Neutral interactions were considered important to note as they were indicative of the majority of interactions occurring among actors where no obviously negative or positive tone was provided. Without considering neutral interactions, the existence and number of connections among actors would have been misrepresented. Due to the variability in the degree of specificity used by interviewees in identifying and describing interactions, actors were coded according to broad sectors (*n* = 8). The aggregation of interaction data into sectors allowed for an analysis of the proportion of interactions (positive, neutral, and negative) reported among sectors. Where network data were analyzed using broad sectors, the number of interactions reported were aggregated and used in the analysis. For example, if there were 10 interviewees within the forestry sector, and collectively they indicated interacting in a positive way with actors within the authorities sector 25 times, the strength of the tie between forestry and authorities for positive interactions would equal 25. This approach to tie aggregation was used consistently throughout the analysis. The original social network data was also retained and coded by geographic scale (local to international) to make possible multi-level analysis of the reported interactions. Local scale was defined as site-specific actors (e.g., a business located within a city); regional was defined as actors that are sub-national but have a broader geographic reach than a site-specific actor (e.g., small-scale forestry owners); national scale was defined as actors with a national (Swedish) reach (e.g., Swedish national authorities); external (national or international) was defined as actors who operate entirely outside of Sweden (e.g., Model Forest in Canada); and the international scale was used for actors who operate at a broader-than-national scale with some reach within Sweden (e.g., EU actors). In the tables, the category of local is sub-divided into two categories: Local 1, which refers to actors within close geographical proximity (the individual municipality), and Local 2, for actors at the local level but potentially situated elsewhere (such as the category reindeer husbandry units in general).

Interactions among broad sectors of land users and managers were investigated using UCINET 6 and Netdraw (Analytic Technologies). Positive, neutral, and negative interactions were recorded in separate matrix files in MS Excel. Matrices are used to organize interaction data for analysis, with the interviewees and the actors they identify listed in identical order in the first row and in the first column of the matrix. A binary approach was used with the original data to create matrices: where an interaction was reported by an interviewee with another actor, a “1” was placed in the corresponding cell; where no interaction was reported, a “0” was placed in the cell. When data were aggregated to the sector scale, the sum of interactions reported by a sector with another sector was placed in the cell (instead of a “1”). Interviewees’ reported interactions at the sector scale were used in an analysis of land user interactions. Density of interactions (total number of ties among sectors divided by total possible ties) was calculated with valued ties, where values indicated the number of times an interaction was reported (Hanneman and Riddle 2005). Density provides insights into how tightly a network is linked. While density cannot be used to compare networks of different sizes (i.e., different numbers of actors), it can provide an indication of differences of types of interactions (positive, neutral, and negative) within cases.

Normalized degree centrality for broad sectors of land users was measured, using directed ties, to identify: (i) the degree to which the sectors in each network were equally mentioning interactions; and, (ii) those actors (sectors) that reported the most interactions with other sectors. Normalized degree centrality is an indicator of the number of interactions a single actor either reported with others (out-degree), or the number of interactions others report with the actor (in-degree). The higher the degree centrality value, the larger the proportion of total interactions the actor is involved in. The use of normalized data allows comparisons among positive, neutral, and negative networks. The use of directed ties provides information about who reported each tie. Using directed ties allows an investigation of how many actors an interviewee, or an aggregated group of interviewees, has reported interactions with (out-degree centrality). It also provides an understanding of how many times other actors, or aggregated groups of actors, indicate interactions with an interviewee (in-degree centrality). Including the quality of the interaction (positive, neutral, or negative) increases the richness of this investigation considerably. Degree centrality was calculated using the Freeman method (Freeman et al. 1991). The group centrality measure was used in UCINET; this measure was designed to manage data from individual nodes (in this study, interviewees) that has been aggregated to a group level (Borgatti et al. 2002).

Cross-level interactions were analyzed using the relational table contingency analysis function in UCINET 6 (Hanneman and Riddle 2005). This analytical procedure tests the differences between the expected and observed frequencies of interactions among actors at various scales for the positive, neutral, and negative networks. For this analysis, all actors within each study site were aggregated by the scale at which they operate (local to international) as the intention was to understand to what degree cross-level interactions were occurring, whether the frequency of reported cross-scale interactions was different from what might occur randomly, and whether there was a tendency for specific scales to interact. The analysis compared observed frequencies of negative, neutral, and positive interactions to the mean expected densities of 10 000 random networks using the same number of scales and interactions and provided a measure of statistical significance for deviation of observed ties to that of expected (randomly generated) ties at the 95 % confidence level.

The model forest ego network, i.e., the network of actors the model forest interacts with, was visualized using NetDraw (Analytic Technologies) with “nodes” representing individual actors (rather than aggregated sub-sector or sector groups). Ties between two nodes indicated a reported interaction, and the arrow originated with the reporting node. The network was arranged in space using the multi-dimensional scaling algorithm in NetDraw that places nodes according to the similarity among interactions (Borgatti and Everett 1997).

To illustrate results of the social network analysis relevant quotes from interviews are presented, excerpted from the full transcripts of interviews and translated from the original Swedish by the authors.

## Results

### Tie Number and Type

Total reported interactions were predominantly neutral; 72 and 67 % of all ties were deemed neutral in Storuman and Vilhelmina, respectively. The next most prevalent type of tie was positive (18 and 21 %, for Storuman and Vilhelmina, respectively), with negative interactions representing the least often reported interaction type (10 % for Storuman and 12 % for Vilhelmina). At the sector level the number of interactions reported per sector was highly correlated with the number of interviewees per sector (*r* = 0.82; df = 4; *p* = 0.004); however, due to our analytical focus on the relative proportion of interactions (positive, neutral and negative), this correlation is not cause for concern (Table 1).

**Table 1:** Number of interviewees and reported interactions per sector

Sectors	Number of interviewees Storuman	Number of reported interactions Storuman	Number of interviewees Vilhelmina	Number of reported interactions Vilhelmina
Authorities in the different sectors (for instance municipality, county administrative board)	4	97	3	73
Forestry (for instance sawmills)	13	199	14	238
Reindeer husbandry (reindeer husbandry units)	2	49	3	67
Tourism (mainly small-scale companies)	6	69	7	109
Nature conservation	1	9	1	26

### Interactions Within and Among Forest Use Sectors

To gain an understanding of interactions at the broad sector level, in- and out-degree centrality scores were calculated for each sector using the aggregated interaction data within all positive, neutral, and negative interaction networks (Tables 2, 3). Focus is placed on: (1) the differences in in-degree centrality (i.e., the normalized number of interactions others reported with specific sectors) *among sectors*; and (2) the differences in out-degree centrality (i.e., the normalized number of interactions a sector reported with others) for positive, neutral, and negative networks *within sectors*.

**Table 2:** Storuman broad sector actor centrality scores

Sector (*n*)	Positive interactions		Neutral interactions		Negative interactions	
	In-degree^a^	Out-degree	In-degree	Out-degree	In-degree	Out-degree
Authorities (32)	0.048	0.058	0.090	0.138	0.021	0.021
Forestry (69)	0.026	0.105	0.046	0.303	0.02	0.111
Infrastructure (14)	0.019	0	0.039	0	0.005	0
Nature conservation (9)	0.005	0.005	0.038	0.019	0.019	0.005
Other (general)^b^ (23)	0.030	0	0.070	0	0.01	0
Other (hydro, wind, mining, ag)^b^ (10)	0.014	0	0.033	0	0.009	0
Reindeer husbandry (10)	0.043	0.047	0.066	0.066	0.014	0.094
Tourism (56)	0.012	0.036	0.072	0.187	0.018	0.006

^a^Centrality measures are based on binary data (aggregated to the broad sector of land user scale) and do not include self-ties

^b^Sectors not represented by interviewees in the study, but identified by respondents as impacting their land use

**Table 3:** Vilhelmina broad sector actor centrality scores

Sector (*n*)	Positive interactions		Neutral interactions		Negative interactions	
	In-degree^a^	Out-degree	In-degree	Out-degree	In-degree	Out-degree
Authorities (34)	0.069	0.037	0.106	0.164	0.048	0.048
Forestry (60)	0.043	0.099	0.031	0.395	0.031	0.049
Infrastructure (34)	0.037	0	0.069	0	0.011	0
Nature conservation (11)	0.009	0.043	0.047	0.038	0.019	0.024
Other (general)^b^ (23)	0.035	0	0.070	0	0.025	0
Other (hydro, wind, mining, ag)^b^ (12)	0.005	0	0.024	0	0.019	0
Reindeer husbandry (6)	0.051	0.046	0.051	0.078	0	0.101
Tourism (40)	0.016	0.099	0.038	0.093	0.016	0.016

^a^Centrality measures are based on binary data (aggregated to the broad sector of land user scale) and do not include self-ties

^b^Sectors not represented by interviewees, but identified by respondents as impacting their land use

For Storuman, interviewees in the authorities, forestry, nature conservation, reindeer husbandry, and tourism sectors reported interactions with other sectors. Other sectors (e.g., infrastructure, hydro, wind, mining, and agriculture) were also identified by interviewees but were not themselves interviewed as part of the empirical material, and interactions are thus identified only from participating sectors. In-degree centrality was similar across sectors for both positive and negative interactions; no one sector stood out as overwhelmingly positive or negative in reported interactions. However, the authorities and reindeer husbandry sectors appeared more central (i.e., more interviewees reported interactions with these sectors) than others in the positive interaction network. When considering out-degree centrality within each sector, reindeer husbandry was the only sector that reported substantially more negative interactions than positive with others (Table 2). This can be explained by the small size of the sector (few active people and economically small although culturally important) requiring large land areas; reindeer husbandry is acutely impacted by many other sectors. One interviewee in reindeer husbandry explained his experience of negative interaction by explaining that they are, in comparison with other sectors with more clearly demarcated land use areas, seen as those who “want to stop development” (Reindeer Husbandry 1, Storuman). In contrast, and potentially also related to the nature of their sector in the study areas (relatively small but with little impact neither from nor on other sectors), tourism in particular reported very few negative interactions with other sectors. As a tourism entrepreneur in Storuman put it, “We have very good contacts locally … with… all actors, which is very positive and very important to us” (Tourism 5, Storuman).

In Vilhelmina, as in Storuman, the majority of interactions were reported as neutral (Table 3). In-degree centrality in the Vilhelmina case showed a higher centrality value for authorities for both positive and negative interactions (Table 3). In the case of authorities, many positive interactions were reported by other categories of actors, for instance, “the collaboration with the municipality has been good” (Tourism 2, Vilhelmina). Similarly, a forestry actor remarked that cooperation works well “with the Forest Agency … because … they… want to have a good collaboration” (Forestry 14, Vilhelmina). Conversely, more negative interactions than positive were reported for the other resource users and nature conservation sectors. However, the interactions reported with reindeer husbandry were in this case entirely positive or neutral (Table 3). As an example of the neutral stance expressed toward reindeer herding, an actor in forestry noted their requirement to “consult with the [reindeer husbandry units] on all logging” (Forestry 14, Vilhelmina). A discrepancy also existed between in- and out-degree positive interaction centrality scores for forestry and tourism, where these sectors reported many positive interactions with others, but others reported relatively few positive interactions with them. For forestry, this may be related to it being a major land use with a large impact on other land uses. Interviewees within the forestry sector noted, for instance, positive interactions with reindeer husbandry, and that the system of consultations with reindeer husbandry “works well…they are flexible and try to respond quickly, and we on the other side, we are trying to do what we can to meet their…needs” (Forestry 14, Vilhelmina).

Also in Vilhelmina, in terms of out-degree centrality, interviewees from the reindeer husbandry sector had a greater tendency than others to report negative interactions (rather than overwhelmingly reporting neutral or positive interactions, as was the case for other sectors) (Table 3). As previously, examples of negative interaction with other sectors highlight that reindeer husbandry, due to its land use interests over large areas, is seen as limiting development in other sectors. For instance, “We have to say no to developments in society affecting reindeer husbandry … [such as] wind power, forestry” (Reindeer Husbandry 3, Vilhelmina). Interviewees in reindeer husbandry both in Vilhelmina and Storuman also did not perceive consultations with forestry as positive; rather, they noted that they have limited possibilities of influencing forestry due to that the interactions are consultations only and do not, for instance, include any veto possibility. Regarding the interaction with the forestry sector specifically, interviewees in reindeer husbandry highlight well-known problems of reindeer husbandry not being able to influence levels of logging:

[Consultations with forestry are] the kind of meetings that … are not empowering … You are going there, and you know that there is not much you can do, you get to talk about it, but then it will not be much more than that. (Reindeer Husbandry 1, Vilhelmina)

### Cross-Level Interactions

The analysis of the interview data highlighted that interactions were not only local, but occurred across a range of scales. A comparison was made of the frequency of reported interactions to the expected interactions within and across scales for positive, neutral, and negative networks.

In both locations, a trend of higher proportions of negative interactions across scales than within (Tables 4, 5) was illuminated, with a mean proportion of observed/expected frequency of 1.41 within scales and 2.19 across scales for Vilhelmina, and 2.62 within scales and 3.16 across scales for Storuman. For positive and neutral interactions, the difference in observed and expected frequencies within scales as opposed to across scales was less pronounced.

**Table 4:** Frequency of observed/expected reported negative, neutral, and positive interactions among scales for Storuman

	No discernible scale	Local 1	Local 2	Regional	National	External (national/international)	International
Negative interactions^a^							
No discernible scale	0	0	0	0	0	0	0
Local 1	5.91	2.28	0	0	2.78	0.93	11.81
Local 2	0	0	0	0	0	0	0
Regional	0	1.77	0	0	0	0	0
National	1.17	0.35	0.56	0	2.96	0	0
External (national/international)	0	0	0	0	0	0	0
International	0	0	0	0	0	0	0
Neutral interactions^b^							
No discernible scale	0	0	0	0	0	0	0
Local 1	1.21	1.55	1	4.16	1.59	1.95	3.47
Local 2	0	0	0	0	0	0	0
Regional	1.56	3.7	2.24	1.82	2.9	0.86	0
National	1.23	1.45	1.97	2.25	2.12	0.34	0
External (national/international)	0	0	0	0	0	0	0
International	0	0	0	0	0	0	0
Positive interactions^b^							
No discernible scale	0	0	0	0	0	0	0
Local 1	1.75	2.24	1.68	0	2.89	0.97	3.07
Local 2	0	0	0	0	0	0	0
Regional	0	0.92	0	0	3.85	0	0
National	0.61	1.45	0.29	1.28	2.82	0	0
External (national/international)	0	0	0	0	0	0	0
International	0	0	0	0	0	0	0

^a^No statistically significantly difference between patterns of observed and expected interactions (95 % confidence level)

^b^Pattern of observed interactions significantly different than expected (95 % confidence level)

**Table 5:** Observed/expected frequency of reported negative, neutral, and positive interactions among scales for Vilhelmina

	No discernible scale	Local 1	Local 2	Regional	National	External (national/international)	International
Negative interactions^a^							
No discernible scale	0	0	0	0	0	0	0
Local 1	1.08	1.29	0.34	4.2	0.38	0.56	1.4
Local 2	3.99	2.35	1.29	3.09	3.52	0	0
Regional	4.99	3.36	0	0	1.76	0	0
National	1.7	0.19	2.11	0	1.64	0	0
External (national/international)	0	0	0	0	0	0	0
International	0	0	0	0	0	0	0
Neutral interactions^a^							
No discernible scale	0	0.75	0	0.79	0.4	0	0
Local 1	0.75	2.04	0.64	1.86	1.09	0.53	1.99
Local 2	0	0.64	0	0.73	1	0	0
Regional	0.79	1.86	0.73	2.72	1.39	0.41	2.04
National	0.4	1.09	1	1.39	1.68	0.09	0
External (national/international)	0	0.53	0	0.41	0.09	0	0
International	0	1.99	0	2.04	0	0	0
Positive interactions^b^							
No discernible scale	0	0	0	0	0	0	0
Local 1	1.3	2.43	1.41	2.01	1.26	0.34	2.51
Local 2	0	0.2	0.77	0	0.84	1.23	0
Regional	0	3.52	0	0	4.21	3.08	0
National	1.02	0.23	0	0	1.71	0	0
External (national/international)	0	0	0	0	0	0	0
International	0	0	0	0	0	0	0

^a^No statistically significantly difference between the patterns of observed and expected interactions (95 % confidence level)

^b^Pattern of observed interactions is significantly different than expected interactions (95 % confidence level)

In Storuman, of particular note is the reported interactions of local scale respondents (Local 1 in the table). These respondents identified a very high number of negative interactions with international actors (more than 11 times the expected frequency based on the mean of 10 000 random networks). There was also a relatively low frequency with which national scale respondents identify negative interactions with local actors (Table 4). Both the neutral and positive interaction frequencies in Storuman were statistically significantly different than the statistically expected frequencies. Positive interactions with national scale actors were higher than expected for all scales of respondents that reported interactions. Mid-scale (regional) actors reported few negative interactions with other scales, some positive interactions at the local and national level, and many neutral interactions across most other scales (Table 4).

Responses separated by scale in Vilhelmina further provided some interesting insights into cross-level interactions. For instance, local scale respondents reported lower than expected frequencies of negative interactions (based on the mean distribution of interactions of the hypothetical networks used for expected frequencies). Many interviewees thus expressed the positive role of local interactions. For instance an interviewee in the tourism sector noted:

The airport in Vilhelmina works very well…The municipality also, they help with procurement of everything, flights, and everything. It works well. The local actors here in the village … you buy some extra groceries and gasoline in the local stores, and then borrow or rent scooters from each other—from these other actors, you buy ski tickets and even book hotel rooms of one another and such. For example, there is a local electrician that we hire, and someone who ploughs … You are connected to each other (Tourism 5, Vilhelmina).

However, respondents reported higher than expected negative interactions with national scale actors (Table 5).

The pattern and frequency of positive interactions were statistically significantly different from the patterns and frequencies expected that is, the positive interaction data could not occur randomly and thus has some special significance. The regional scale respondents reported more frequent positive interactions than expected for all scales where interactions occurred. Mid-scale (regional) actors reported more frequent than expected negative interactions with local and national actors; however, these values were not statistically significant and regional actors were also interacting positively with the same scales and neutrally across all scales—from local to international (Table 5).

**Fig. 2:**
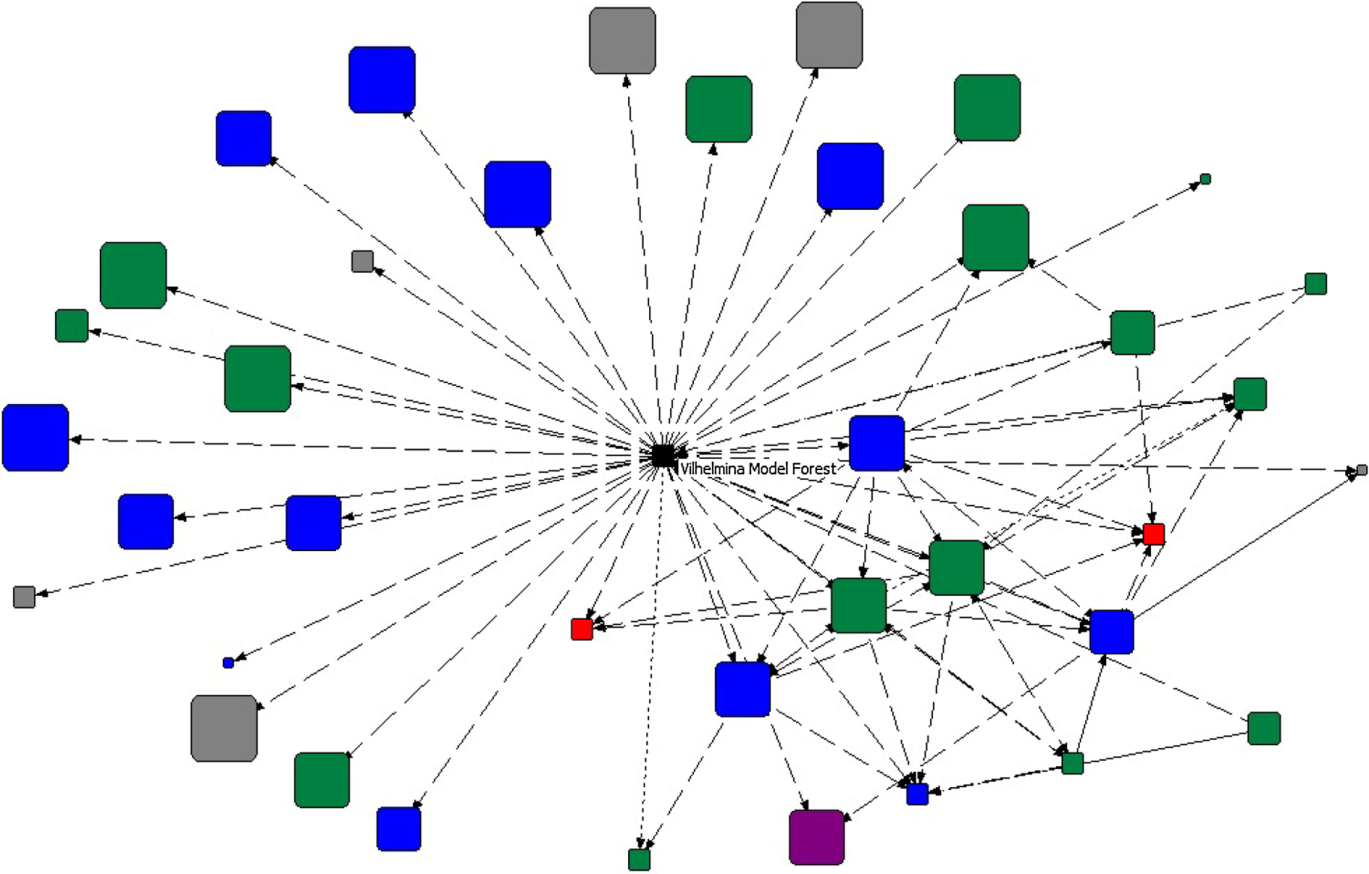
Vilhelmina Model Forest ego network. Positive and neutral interactions are visualized (*dashed lines* represent neutral interactions, *dotted lines* represent positive interactions, and *solid lines* represent more than one interaction type). *Node size* represents scale, from local to international (with the smallest nodes indicating scale could not be determined). *Colors* are indicative of broad sectors: *blue* authorities; *green* forestry; *red* reindeer husbandry; *purple* nature conservation; *gray* other

**Fig. 3:**
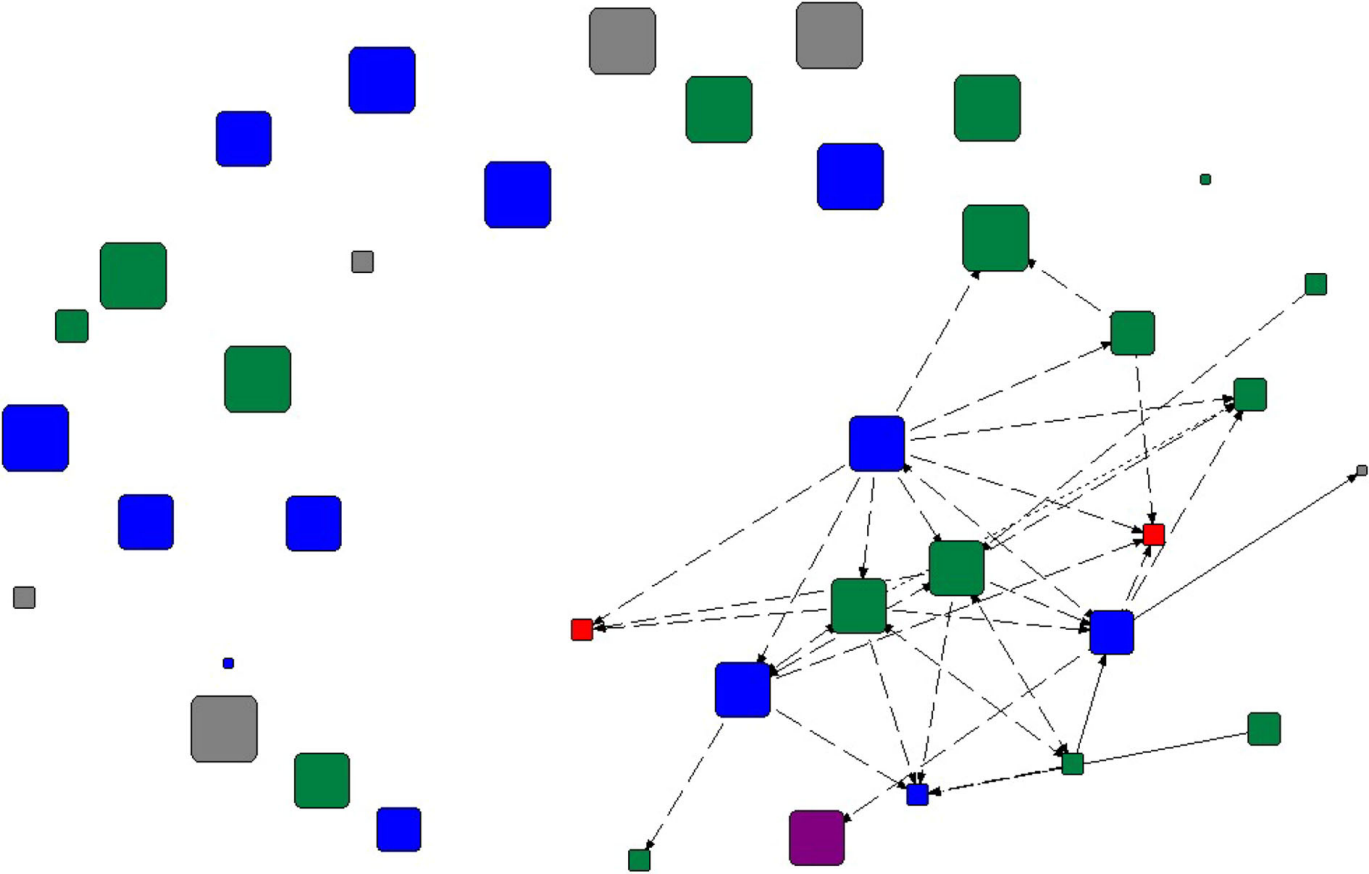
Effect of Vilhelmina Model Forest removal from ego network. Positive and neutral interactions are visualized (*dashed lines* represent neutral interactions, *dotted lines* represent positive interactions, and *solid lines* represent more than one interaction type). *Node size* represents scale, from local to international (with the smallest nodes indicating scale could not be determined). *Colors* are indicative of broad sectors: *blue* authorities; *green* forestry; *red* reindeer husbandry; *purple* nature conservation; *gray* other

A particular role, akin to some regional actors, could be seen also for the model forest. The Vilhelmina Model Forest plays a specific role in that it aims to provide a forum for discussion and local engagement on forest use, and in that to interact with and support cooperation among various forest users. The model forest thus interacts with several sectors: forestry, authorities, nature conservation, and others (Fig. 2). Most of these interactions were neutral or positive, and the interviewee reported interacting with 38 actors at scales from local to international—approximately half at the national and international scale, and half at the regional scale or smaller. With the removal of the model forest from this network, 21 actors became disconnected from the model forest network (including certain Swedish authorities, a forest owner association and reindeer husbandry units), and 19 of those actors became disconnected from the entire Vilhelmina network (including other model forests, and forestry authorities outside Sweden); most at the national or international scale (Fig. 3).

The potential for the model forest as a bridge between regional and smaller scale actors with national and international actors can thus be identified from the network analysis. However, important to note is that the forestry, reindeer husbandry, and nature conservation sectors were still connected and interacting with one another even without the model forest. While these interactions may thus occur regardless of the existence of the model forest, cross-level and cross-sector interaction may be extended through the model forest, as indicated by the model forest ego network.

## Discussion and Conclusion

This study describes and analyses the existence of user interactions and impacts in the forest-based sectors as well as how these may extend to regional, national, and international levels. It illustrates many of the interactions between sectors that have been highlighted in literature on northern Sweden. These include the relationship between forestry and reindeer husbandry, where forestry has fairly consistently (since the 1970s) reported a positive or neutral relation to reindeer husbandry in relation to required consultation meetings, while reindeer husbandry has instead consistently reported negative interactions with forestry, in particular related to being a smaller sector and being unable to limit the extent of forestry (Brandt and Lundberg 1987; e.g., Keskitalo 2008a). This can be seen as a consequence of the basic situation for reindeer husbandry, with some 2500 reindeer herders active over about the same area as large forest companies, small-scale forest owners and numerous other interests (in total up to some 60 000 owners)—as could be expected over an area of more than a third of Sweden (cf. Sandström and Widmark 2007; Keskitalo 2008a; Government Offices of Sweden 2013). In this respect, the study illustrates the importance of explicit consideration of negative ties in network analysis and in order to understand limitations in the extent to which network linkages may support governance, as well as the extent to which local governance conflicts may in fact manifest as a result of property rights arrangements determined at a higher level.

So far, there has been little focus on negative interactions in social network literature; however, those who have investigated negative interactions suggest that natural resource governance efforts can be jeopardized as a result. For example, Robins et al. (2011) state that a fragmented “macroculture”—disputed or conflicting priorities among actors—leads to more negative interactions and may place the effectiveness of governance in jeopardy (Jones et al. 1997; Robins et al. 2011). Tucker (2004) cautions that a lack of coordination and tensions among actors at different scales can compromise natural resource governance efforts. From the results of this study, potential impacts of perceptions of negative interactions can be identified. For example, there exists a disconnect between some sectors, such as reindeer husbandry and others (forestry, authorities) in Storuman and forestry and tourism in Vilhelmina, in that these sectors are perceiving interactions with others differently than those sectors perceiving interactions with them. This finding emphasizes the challenges that may be associated with initiating cooperation among sectors and incorporating multiple levels into any network governance approach. The explicit consideration of multiple tie types (i.e., the positive, neutral or negative tone of reported interactions) and cross-level interactions is important when studying natural resource management, as neglecting these elements may result in an over- or under-estimation of the cohesiveness of the network and a misrepresentation of the role of networks at local or regional levels. The investigation of negative interactions and natural resource governance offers a fruitful area for further research, and could potentially be used in order to identify such challenges also in cases where existing conflicts are not well researched.

However, while providing insights into the density of connections and into large neutral and positive as well as multi-level connections of interactions, this study also illustrates positive cross-level interactions and implications for forest use and management. Of particular interest from a capacity perspective are the linkages between regional actors and other scales (i.e., as potential cross-level brokers following Ernstson et al. (2010). Bridging actors are identified as important in the success of natural resource governance (Lauber et al. 2008), linking local and broad-scale actors (Ernstson et al. 2010). Regional bridging actors can facilitate collective action where it might not otherwise occur (Bodin and Crona 2009). The results from the analysis of positive, neutral, and negative connections across levels indicate that, in both case studies but especially in Storuman, the regional actors were involved in more positive and neutral interactions with other levels of actors (e.g., local, national). More negative interactions than expected between local and national actors further highlight tensions among levels and the potential role of regional actors to act as cross-level brokers in any collaborative governance arrangements. Cooperation arenas such as here in the case of the model forest also serve as a means of constituting a bridge between some of the different (heterogeneous) actors, in particular to actors at other levels and thereby potentially enriching the connections of certain local actors. The existence of bridging actors, such as the Vilhelmina Model Forest, also plays a potentially important broker role in providing linkages across levels.

## Footnotes

1 Large variations in how the reindeer husbandry area is calculated exist in different publications. For instance the Swedish Saami Parliament (2013) notes that reindeer husbandry is practiced over 52 % of Sweden’s area, as does Statistics Sweden, noting about 50 % (2008). However, Statistics Sweden notes that this is built on a schematic assessment where not all land in the winter grazing area is attributed for reindeer husbandry. The largest areas for reindeer husbandry are found in Norrbotten, Västerbotten and Jämtland where some 80 % can be counted as reindeer grazing areas. An assessment that targets these counties may approximate closer to 40 % of Sweden’s area (Sandström and Widmark 2007; see also Bostedt et al. 2003; Widmark 2009). Assessments around a third of the Swedish land area are found in Government Offices of Sweden (2013) and Jernsletten and Klokov (2002).

2 It should also be noted that persons other than those actively involved in reindeer husbandry own reindeer: the number 2500 active reindeer herders reflect activity, while the term “reindeer owner” reflects all those listed in reindeer husbandry unit reindeer registers. In total do about 3500 Saami and about 1000 non-Saami own reindeer in Sweden (Government Offices of Sweden 2013). Eight of the 51 reindeer husbandry units that exist in the country are so called concessionary, in which non-Saami owning agricultural properties in the northernmost county, Norrbotten, in specific cases have the right to own but not themselves manage reindeer (Ministry of Agriculture 2001; Government Offices of Sweden 2013). There is thus a relatively high percentage of non-Saami reindeer owners.
